# Metabolomics and Molecular Networking to Characterize the Chemical Space of Four *Momordica* Plant Species

**DOI:** 10.3390/metabo11110763

**Published:** 2021-11-08

**Authors:** Anza-Tshilidzi Ramabulana, Daniel Petras, Ntakadzeni E. Madala, Fidele Tugizimana

**Affiliations:** 1 Department of Biochemistry, University of Johannesburg, Auckland Park, Johannesburg 2006, South Africa; ramabulanaanza@gmail.com; 2 CMFI Cluster of Excellence, Interfaculty Institute of Microbiology and Medicine, University of Tubingen, Auf der Morgenstelle 28, 72076 Tubingen, Germany; daniel.petras@uni-tuebingen.de; 3 Department of Biochemistry and Microbiology, Faculty of Sciences, Agriculture and Engineering, University of Venda, Private Bag X5050, Thohoyandou, Limpopo 0950, South Africa; Ntakadzeni.Madala@univen.ac.za; 4 International Research and Development Division, Omnia Group, Ltd., Johannesburg 2021, South Africa

**Keywords:** GNPS, LC-MS, metabolomics, molecular networking, *Momordica*, natural products

## Abstract

*Momordica* plant species (*Cucurbitaceae*), have been used for centuries in traditional medicine and for nutritional purposes. Plants from this family are thus claimed to be phytochemically rich, representing an inexhaustible source of natural products. However, the chemical space of these *Momordica* species has not yet been fully decoded, and due to the inherent complexity of plant metabolomes, the characterization of the *Momordica* phytochemistry remains challenging. Thus, in this study we propose the use of molecular networking to unravel the molecular families within the metabolomes of four *Momordica* species (*M. cardiospermoides*, *M. balsamina*, *M. charantia* and *M. foetida*) and highlight the relevance of molecular networking in exploring the chemotaxonomy of these plants. In silico annotation tools (Network Annotation Propagation and DEREPLICATOR) and an unsupervised substructure identification tool (MS2LDA) were also explored to complement the classical molecular networking output and integration using MolNetEnhancer within GNPS. This allowed for the visualisation of chemical classes and the variety of substructures within the molecular families. The use of computational tools in this study highlighted various classes of metabolites, such as a wide range of flavonoids, terpenoids and lipids. Herein, these species are revealed to be phytochemically rich plants consisting of many biologically active metabolites differentially distributed within the different species, with the metabolome of *M. cardiospermoides* dereplicated in this paper for the first time.

## 1. Introduction

*Momordica* species fall within the Cucurbitaceae family commonly known as the pumpkin, cucumber, gourd, or melon family [1]. The *Momordica* genus comprises 59 species, geographically distributed across the globe (47 in Africa, 12 in Australia and Asia) [2,3]. *Momordica* species are cultivated not only for their nutritional purposes but also for traditional use in the treatment of various ailments [4]. These include diabetes-related conditions [5], cancer [6] and inflammatory diseases [7]. Other activities that have been reported for species within this family are anti-obesity [8], antioxidant [9] and potential anti-HIV activities [10]. These activities can be attributed to the diverse phytochemical composition of these plants. *Momordica* species have been reported to contain various health-promoting compounds such as cucurbitane triterpenoids, saponin glycosides, chlorogenic acids and flavonoids [1,11,12,13,14]. 

These health benefits and bioactivities of *Momordica* species point to a diverse and rich source of numerous bioactive metabolites. Although studies have provided some insights into the phytochemistry of these plants, there are still knowledge gaps regarding the chemical space of *Momordica* plants. Currently, the metabolomes of *M. charantia*, *M. balsamina* and *M. foetida* are partially profiled, whilst that of *M. cardiospermoides* has yet to be reported. Thus, there are still ‘dark matter’ in regard to chemical identities and compositions of *Momordica* extracts; a lack of comprehensive description of metabolomes of *Momordica* species. Furthermore, the relationship between the whole metabolome of *Momordica*-derived remedies and their biological effects is still enigmatic. Metabolomics, a multidisciplinary *omics* science, with emerging computational metabolome mining tools, offers unique opportunities to provide a comprehensive qualitative and quantitative description of all metabolites in a biological system [15,16]. Recently, hyphenated metabolomics techniques such as HPLC-MS have been employed in the dereplication of complex secondary metabolites in *B. pilosa* [17] and in *Coccinia* plants [18]. In combination with developing computational tools, a broader view of metabolomes has been achieved as in maize plant treated with biostimulants [19] and comprehensive annotation of flavonoids in Chrysobalanaceae plants was achieved [20]. The computational tools and strategies make it possible to decompose complex metabolite mixtures into substructures and chemical class information, thereby aiding in the annotation of known and unknown metabolites [21,22]. As such, metabolomics is an indispensable approach to decode and comprehensively characterize the metabolite profiles/phytochemistry of *Momordica* species. 

Thus, in this study, a liquid chromatography–mass spectrometry (LC–MS)-based untargeted metabolomics approach, involving computational strategies, was applied to characterize the chemical space of four *Momordica* species occurring in the Venda region of South Africa: *M. cardiospermoides*, *M. balsamina*, *M. charantia* and *M. foetida*. Molecular networking (MN) approaches, in the Global Natural Products Social Molecular Networking (GNPS) ecosystem [23] were applied to process and analyze the generated spectral data. The workflows used herein build on the fundamentals that metabolites within complex mixtures are diversified forms originating from the same building blocks (substructures) [21]. Molecules sharing similar MS^2^ spectra were thus grouped into molecular families using MS-Cluster algorithm while also matching them to MS^2^ spectral libraries (e.g., NIST, Respect and MassBank). The annotation of a metabolite in such molecular families then facilitated annotation of its structurally related neighbours [22,24,25]. Other tools, such as in silico annotation tools (network annotation propagation and DEREPLICATOR), and an unsupervised substructure identification tool (MS2LDA) were also explored to complement the classical molecular networking output and integration using MolNetEnhancer within GNPS. This would allow for the visualisation of chemical classes and the variety of substructures within the molecular families [25]. Computational metabolomics holds great promise in increasing identification coverage of MS/MS spectra and improving biochemical insights derived. 

## 2. Results and Discussion

Untargeted metabolomics spectral data (from the aqueous-methanol extracts of leaves of the four *Momordica* species) was obtained using an LCMS quadrupole time-of-flight (Q-TOF) in both ionisation modes (ESI (+/−). The spectral data were acquired using data dependent acquisition (DDA) protocol, automatically obtaining MS^2^ fragmentation spectra for all precursor ions above a defined threshold (Section 3.3). A visual inspection of the chromatographic space points to the presence (in the extracts) of a wide range of metabolites of different polarities, which are differentially distributed across the *Momordica* species (Figure S1). Furthermore, in the principal component analysis (PCA) score space, distinct sample groupings were observed (Figure S2A), implying species-dependent differential metabolic profiles. The distinct sample grouping performed was influenced by metabolites indicated in the PCA loadings plot (Figure S2B). The HCA model (Figure S2C), computed based on the measured (processed) spectral data, also revealed that the metabolome of *M. balsamina* clusters different from the other three species, with *M. foetida* and *M. charantia* being more similar. In a previous study on three *Momordica* species (*M. foetida*, *M. charantia* and *M. balsimina*), these species were described to share similar flavonoid profiles with *M. charantia* and *M. balsamina* being more chemotaxonomically related [14]. However, here the chemometric models computed indicates that the four metabolomes under study are chemotaxonomically distinct, especially that of *M. balsamina*. To illuminate the major chemical classes and explore the metabolomes of the four *Momordica* species, these spectral data were subjected to molecular networking (in GNPS platform), enabling a broad overview of molecular information that can be inferred from MS/MS data. Network analysis, in silico annotation and substructure annotation job links are provided in the supplementary materials. 

### 2.1. Major Chemical Classes of Momordica Metabolomes 

**Figure 1:**
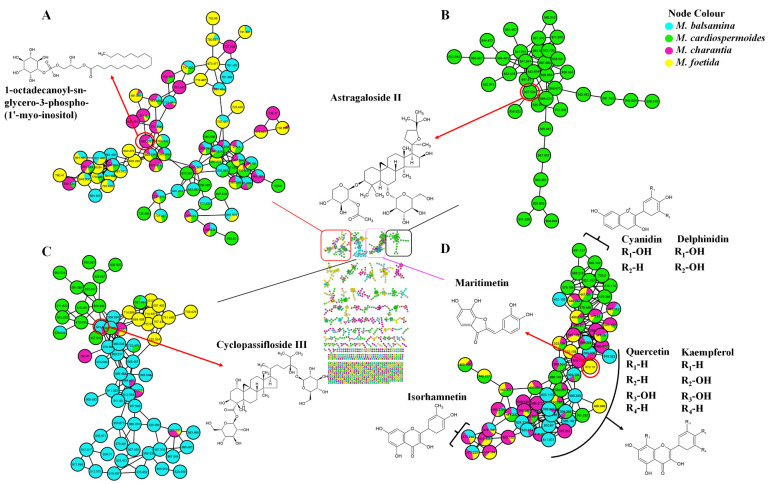
Molecular network of *Momordica* species extracts analysed by liquid chromatography–tandem mass spectrometry using electrospray ionisation in negative mode (centre), with four major metabolite classes identified highlighted here glycerophospholipids (**A**), triterpenoids (**B**), terpene glycosides (**C**) and flavonoids (**D**). Highlighted are also the aglycones/backbones from which the flavonoids identified are derived. Node colours represent the studied *Momordica* species and the respective MS^2^ spectral counts indicating presence and absence of metabolites.

Classical molecular networking was firstly applied. The MS-Cluster algorithm was used to evaluate inter- and intra- sample spectra similarities, grouping ions within a predefined mass tolerance into consensus spectra, which are represented as nodes. Structurally related metabolites that share similar gas phase chemistries were grouped into molecular families based on their similarity scores (cosine scores ≥ 0.7) [20]. In the computed MN, 1493 consensus spectra (nodes) were generated, with 1022 clustered into 165 independent molecular families (with a minimum of two nodes connected by an edge) based on GNPS spectral matching. Spectra not clustered into molecular families were represented as self-loop nodes at the bottom of the network (Figure 1 and Figure S3A). In the computed MN (Figure 1), 57 of the nodes were putatively annotated through an automated library spectral matching, providing some insight into the chemical identities of the *Momordica* species but also alluding to the metabolome complexity of these plants and the lack of comprehensive spectral libraries. This analysis of chemical relationships between every MS/MS spectrum, visualising the entire metabolome detected in a sample, revealed structurally related molecular families in *Momordica* species. 

A vast number of lipids could be identified, such as glycerophospholipids (Figure 1A), for example, 1-octadecanoyl-sn-glycero-3-phospho-(1′-myo-inositol) which was identified in three species but not in *M. cardiospermoides*. Glycerophospholipids are lipids with both a phosphoric acid and fatty acid attachment and are classified as heterolipids. These form a large component of cell membranes and have vital functions in cellular physiology [26,27,28]. Other metabolites that are widely distributed in the four species are flavonoids, which clustered into a molecular family (Figure 1D) that assisted in putative annotation of about 28 flavonoids across the four species. Flavonoids are widely distributed in fruits and vegetables and are described as having many health-promoting effects, such as antioxidant [29], anti-cancer [30], and anti-bacterial [31] activities. These are used by plants for growth and among others, protection against oxidative stress and UV-A and UV-B protection [32,33]. Here, the four plant species were highlighted as to be rich in flavonoids which are polyphenolic in structure and biosynthesised through the phenylpropanoid pathway. Although the biosynthesis of flavonoids is conserved in plants, various subclasses arise due to action reductases, isomerases, dioxygenases and hydroxylases [34]. Thus, flavonoids have structurally diverse aglycones as backbones namely, chalcones, flavones, isoflavones, flavanols, flavonols, flavanones and anthocyanidins. These backbones occur in various modified forms through hydroxylation, methylation, and glycosylation by transferases. Occasionally other decorations may attach to the aglycones such as sulphates and aliphatic acids [34,35,36]. In the current study, flavonoids identified had aglycones that fall into 4 subclasses which mostly were flavonols (quercetin, kaempferol and isorhamnetin), two anthocyanins (cyanidin and delphinidin), one flavanone, one flavone and one aurone flavonoid (maritimetin, structural isomers of flavones) as listed in Table S1 and Figure 1, decorated with various sugar attachments. The flavones and flavanones were observed to cluster separately from other flavonoids deriving from flavonol, anthocyanin and aurone aglycones, which could indicate some fragmentation differences between these subclasses. Interestingly, flavonoids identified were seen to also go through glycoisomerisation as described by [18], as observed for quercetin rutinoside (Table S1), which presented similar pseudo-molecular ions with the same fragmentation patterns. This could suggest a slight shift in the position of the glycosidic bond between the disaccharide that is conjugated to the quercetin aglycone. 

Putative annotation of metabolites that were matched and some unmatched by molecular networking were confirmed based on their accurate mass, which was used to generate molecular formulae that were searched across databases. It is important to note that during MS/MS fragmentation, most flavonoids readily lose their sugar attachments, resulting in aglycones exposure for further fragmentation [14,37]. Interrogation of the spectral network or the flavonoid subcluster (Figure 1D) indicated some spectral signatures within the data, such as sub-clusters within the molecular family based on the nature of aglycone. This could possibly imply that molecular networking could be useful in highlighting the backbone structures of metabolites, thus aiding in their structural elucidation. Specialised flavonoid production was observed across the species, indicated by the node colouring (Figure 1D), which shows the MS^2^ spectral counts indicating presence/absence of these metabolites across the groups [38]. The two observed anthocyanidins (derived from delphinidin and cyanidin, Figure 1D) were only observed in *Momordica cardiospermoides*, indicating that this species differs from the other three species through specialised productions of anthocyanidins. These differences could possibly be useful for taxonomical and biological classification of these species. Moreover, in comparison to the other flavonols (quercetin and kaempferol derivatives), it was observed that isorhamnetin derivatives were the least present with only four identified. These results were consistent with results reported in [14] on three *Momordica* species. Interestingly, *M. foetida* was observed to also have a unique metabolite signature consisting of the only maritimetin or aurone flavonoid identified (Figure 1D). This can also be used in the taxonomical classification of these plant species.

Other metabolites that were identified in the four *Momordica* species were mainly hydroxycinnamic acid derivatives, triterpenoids, terpene glycosides and various lipids (Table S1). As observed in Figure 1B,C *Momordica* species contain a wide range saponins (terpene glycosides and triterpenoids). These are high molecular weight, structurally complex molecules consisting of a triterpene molecule (aglycone, 27–30 carbons), attached to one or more sugar moieties through a glycosidic linkage. The chemical complexity of saponins is described as the gap between understanding the relationship between their structures and the relative pharmaceutical and medicinal properties [39]. Various activities have been attributed to these glycosides, such as anti-diabetic, anti-inflammatory [40,41,42], anti-tumour and anti-bacterial activities [43]. In this study, 19 saponins were identified, including cycloartane triterpenoids. For instance, cyclopassifloside III (Figure 1C) was identified in *M. balsamina* and *M. charantia*. Cycloartane triterpenoids, initially described in *Passiflora edulis*, have been reported to be important secondary metabolites with anti-depressant activities [44]. Furthermore, as it can be inferred from the MN, the four species produce a unique type of triterpenoids. The latter are structurally related, but with structural nuances across the species (Figure 1C). This indicates some chemotaxonomic differences between the species. Although various triterpenoids were identified across the species, *M. balsamina* was found to have the least number of triterpenes. The molecular family with some triterpenoids identified indicated that the four species contain chemically related metabolites that are distributed uniquely across the species (Figure 1C). *M. cardiospermoides* was found to produce distinct triterpenoid glycosides (Figure 1B), clustered in their own molecular family. Some of these were previously described to have biological activities, such as Astragaloside II, which is a cycloartane triterpenoid glycoside; the latter are reported to have some immunostimulatory and anti-cancer properties [45]. The infographic depiction of subtle differences within the molecular families (presence and absence of some metabolites) could be useful in deciphering the chemotaxonomy of plant species. In this study, the classical molecular network computed (Figure 1 and Figure S3A), assisted in the identification of important secondary metabolites, which were previously not described in the *Momordica* species, such as the above mentioned saponins.

### 2.2. Detailed Exploration of the Chemical Space of Momordica Species 

**Figure 2:**
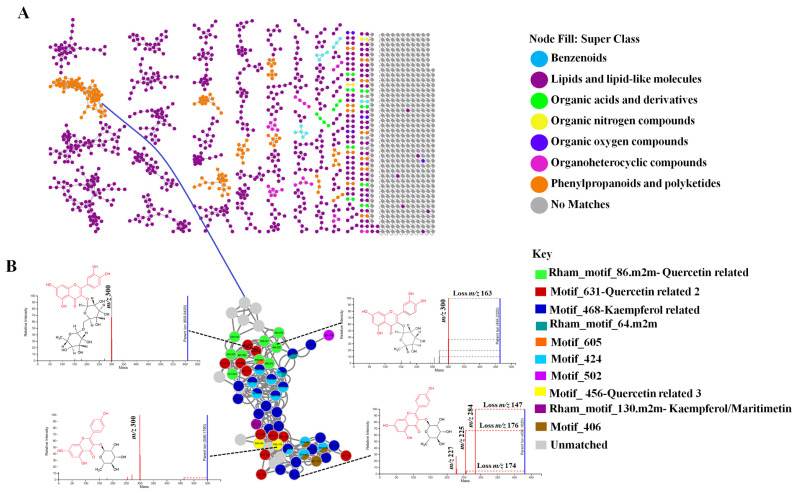
Chemical classification and structural elucidation. (**A**) An enhanced molecular network in which nodes are coloured based on their chemical superclass. The node annotations were based on substructure annotation (MS2LDA), network annotation propagation (NAP) and DEREPLICATOR output. (**B**) Metabolite annotation using NAP and by MS2LDA, the coloured parts of the structures indicate the parts that make up the Mass2Motifs.

To further explore the metabolomes of *Momordica* species, MolNetEnhancer was applied (Figure 2 and Figure S3B). Classically, molecular networking has some limitations, including the dependency on spectral library matching to annotate the generated molecular families [25], and reference spectra in public repositories are still few (approximately 2–5% MS^2^ spectra can be matched to known compounds) [46]. Various other tools are available in GNPS that complement molecular networking, such as in silico annotation tools (network annotation propagation and dereplication) which perform in silico fragmentation of known structures, then searching against chemical databases. The predicted structures are then ranked based on similarity to the experimental masses. MS2LDA can also be used, which applies unsupervised latent Dirichlet allocation (LDA) decomposition to MS/MS spectra, discovering substructures within the molecular families based not only on common fragment patterns but also on neutral losses shared by structural analogs. The substructures identified are termed Mass2Mottifs, which are unsupervised groups of fragments and/neutral losses. Substructure discovery may provide insight into the backbone of metabolites, such as the functional groups, building blocks and/core structures. This is feasible as complex metabolite structures may be synthesised from common biosynthetic pathways, thus sharing the same building blocks [21,47]. 

Thus, the MolNetEnhancer approach integrates result outputs from molecular mining tools (molecular networking and MS2LDA), in silico annotation tools (NAP and DEREPLICATOR) and class annotation through ClassyFire terms to provide a comprehensive visualisation of the chemical space within a metabolome. The class annotations and taxonomy are performed by calculation of the most abundant chemical classes within the molecular families, the putative structures can be organised in chemical taxonomic hierarchical levels such as the superclass level (Figure 2A and Figure S3B) [21,25]. The visualisation of the chemical space of the four *Momordica* species through MolNetEnhancer (Figure 2A and Figure S3B) indicated that these species contained benzenoids, lipids and lipid like molecules, organic acids and their derivatives, organic nitrogen and oxygen compounds, organoheterocyclic compounds, phenylpropanoids and polyketides. Previously, *Momordica* species have been reported to contain a wide variety of compounds, including triterpenoids, saponins, oils, steroids, glycosides, and flavonoids. These compounds were mostly described in *M. charantia*, *M. balsamina* and *M. foetida* [4,14,48,49] but chemical insights on *M. cardiospermoides* are still lacking. The computational tools used herein, assisted in the unravelling of the metabolome of this species, reported here for the first time. Herein, the use of chemical classification into taxonomic hierarchical levels was shown to broaden insights into the metabolomes of complex metabolomes of *Momordica* species. As observation post computational annotation of metabolites indicated from the extracted metabolites, the newly described metabolome of *M. cardiospermoides* is rich in flavonoid glycosides, flavonols, terpen glycosides and triterpenoids (Figure 1).

### 2.3. Pathway Analysis and Relative Quantification of Metabolites in the Four Momordica Species

**Figure 3:**
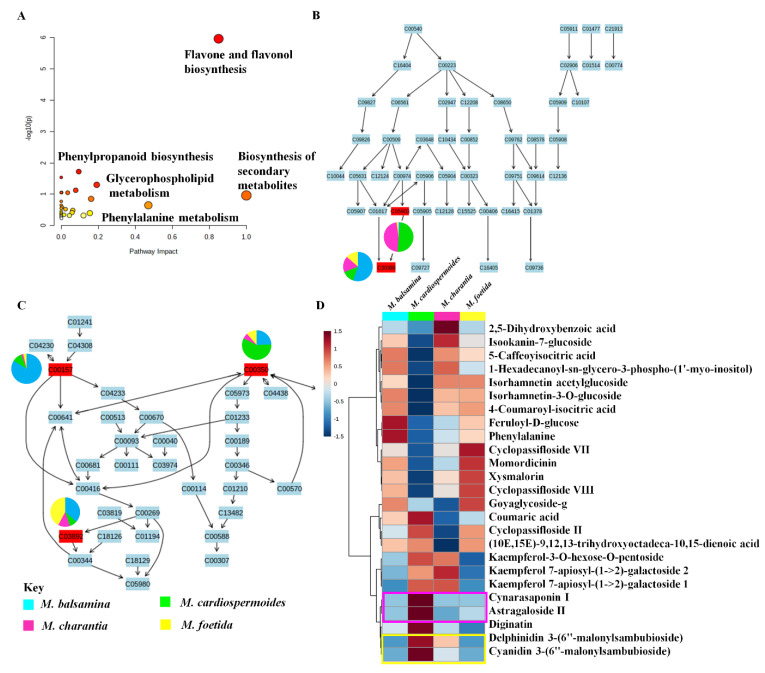
A summary of the metabolism pathways identified in *Momordica* species (**A**). An indication of the flavonoid biosynthesis pathway (**B**). The glycerophospholipid pathway (**C**). The relative quantification of some metabolites identified across the four *Momordica* species, highlighted in yellow are the anthocyanin derivatives abundant in *M. cardiospermoides* and in purple some saponins that are abundant in *M. cardiospermoides* and *M. charantia* (**D**).

Based on pathway over-representation of the differentially abundant metabolites in the four *Momordica* species, 18 biological pathways were the most statistically enriched (Table S2, Figure 3A). These include the glycerophospholipid pathway, flavonoid biosynthesis pathway and the flavone and flavonol biosynthetic pathways. In the flavonoid biosynthesis pathway, two aglycones could be mapped (quercetin and kaempferol), which are backbones to most flavonoids identified in this study. Furthermore, the enriched the glycerophospholipid/phosphoglycerolipid biosynthesis pathway was revealed (Figure 3C), in which the three glycerophospholipids that could be mapped were found to be present across the four species with varying relative concentrations. Glycerophospholipids form major components of membranes and play some roles in signal transduction [50]. Furthermore, a hierarchical clustering heatmapping of normalized data (with autoscaling features, Euclidean distance measurement and Ward clustering method) allowed (relative) quantitative description of these metabolic profiles of the four *Momordica* species. For instance, the relative quantification of the top 25 metabolites (based on t-test/ANOVA) indicated the changing relative concentration of the normalised data set across the species (Figure 3D) [51].

**Figure 4:**
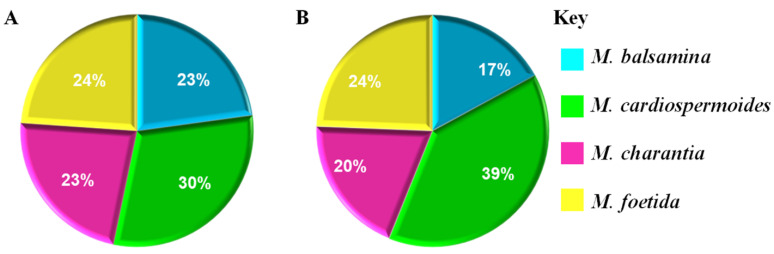
Graphical summary of the presence and absence of metabolites from two major annotated classes. (**A**) indicate the distribution of the annotated flavonoid glycosides and flavonols (phenylpropanoids across the four species. (**B**) show the distribution of triterpenoids and terpene glycosides (lipid and lipid-like molecules) across the four *Momordica* species.

The relative quantification of some metabolites identified across the species, showed *M. balsamina, M.charantia*, and *M. foetida* to only have some subtle differences in (relative) levels of measured metabolites. Distinctively, *M. cardiospermoides* was found to contain the highest levels of the identified anthocyanin flavonoids arising from the delphinidin and cyanidin aglycones. Various studies have reported the importance of anthocyanins in the treatment of prevention of human disease, such as cardiovascular diseases and many other diseases [52]. The comparative concentration of the anthocyanins between *M.cardiospermoides* and the other three species could indicate that *M. cardiospermoides* is a better source of this natural product with important applications (Figure 3D). This species is also observed to have high relative concentration of saponins, such astragaloside II (Figure 3D), which was described above to have important biological activities. *M. cardiospermoides* was also found to contain the lowest relative concentration of phenylalanine, which is an amino acid that forms an important connection between the primary and secondary metabolism leading to a production of metabolites such as phenylpropanoids [53] i.e., coumaric acid, which was relatively abundant. All four species were observed to have differential relative intensities of annotated metabolites, highlighting these species as phytochemically rich. Furthermore, it can be postulated that the observed differential metabolic profiles point to genotypic differences between the four species, also considering the plant adaptations to environmental conditions [54]. Thus, this study evidences that *M. cardiospermoides* is an alternative source of metabolites, which are not found in the other three *Momordica* species (Figure 4). This suggests that *M. cardiospermoides* has a different enzymatic and biochemical machinery for the biosynthesis of these metabolites. These metabolic insights pave a way for future genetics studies to characterize the functional genetic makeup that governs the biosynthesis of metabolites in the *M. cardiospermoides* system. The computational tools used above, namely molecular networking and MolNetEnhancer, with complementary in silico tools provided a semi-automated annotation of the metabolome. This also led to better comprehension of the biology of the four *Momordica* species.

## 3. Materials and Methods

### 3.1. Plant Material

*M. cardiospermoides* (Tshirolwe/Mutititi, −22.896, 30.005), *M. balsamina* (Luvhalani, −22.9064, 29.9644)*, M. charantia* (Tshisaulu, −23.002807, 30.401409) *and M. foetida* (Itsani, −23.00511, 30.41892) leaves were collected from the Venda region, in the Limpopo province of South Africa. These plants were collected in areas that are in close proximity to each other and share similar environmental condition. The leaves were harvested during summer while the plants were in fruit ripening stage. The plant specimens were authenticated through the help of Dr K Magwede using the already deposited specimens within the University of Venda herbarium facility, South Africa. The plants were air-dried at room temperature and crushed (with a blender) to powder form and stored until metabolite extractions. 

### 3.2. Metabolite Extraction and Sample Preparation

Two grams (2g) of the samples were weighed and extracted in 20 mL (1:10 *m*/*v*) of 80% methanol (Romil SpS, Cambridge, UK). The samples were spun overnight in a digital rotisserie tube rotator at 70 rpm. The crude extracts were centrifuged at 5100 rpm in a benchtop fixed angle centrifuge (Thermo Fisher, Johannesburg, South Africa). The supernatants were filtered using 0.22 µm nylon filters into glass vials with 500 µL inserts. At least 5 independent replicates for each sample group were prepared, these were stored at 4 °C until analysis. Quality control (QC) samples were prepared by pooling equal volumes of each sample to assess the quality of the generated data, which were ran alongside experimental samples (Section 3.3).

### 3.3. Liquid Chromatography-Quadruple Time-of-Flight Tandem Mass Spectrometry (LC-MS/MS) 

Extracts were analysed on a liquid chromatography–quadrupole time-of-flight tandem MS instrument (LCMS-9030 qTOF, Shimadzu Corporation, Kyoto, Japan). The chromatographic separation was performed on a Shim-pack Velox C18 column (100 mm × 2.1 mm with particle size of 2.7 µm) (Shimadzu Corporation, Kyoto, Japan) at 55 °C. For all samples an injection volume of 3 µL was used and run using a binary mobile phase gradient which consisted of solvent A: 0.1% formic acid in Milli-Q water (both HPLC grade, Merck, Darmstadt, Germany) and solvent B: Methanol (UHPLC grade, Romil SpS, Cambridge, UK) with 0.1% formic acid. The flow rate was set to 0.3 mL/min throughout the set 53 min gradient with the following separation conditions: 10% B maintained for 3 min, 10–60% B over 3–40 min, from 40–43 min the conditions were maintained at 60% B, then the gradient was changed to 90% B between 43–45 min and maintained at 90% B for 3 min. The gradient was returned to initial conditions between 48–50 min which was followed by a 3-min column requilibration time. The chromatographic effluents were further analysed utilizing the qTOF high-definition mass spectrometer set to acquire negative electrospray ionisation data. The subsequent parameters were set as: interface voltage of 4.0 kV, interface temperature of 300 °C, nebulization and dry gas flow 3 L/min, heat block temperature of 400 °C, DL temperature of 280 °C, detector voltage of 1.8 kV and the flight tube temperature at 42 °C. Sodium iodide (NaI) was used as a calibration solution to monitor high mass accuracy. MS^1^ and MS^2^ (through data dependent acquisition) were generated simultaneously for all ions with an *m/z* range between 100–1000 surpassing an intensity threshold of 5000. Fragmentation experiments were performed using argon as a collision gas at a collision energy of 30 eV with a spread of 5 eV. Quality control (QC), pooled samples were used to condition the LC-MS system and for non-linear signal correction. The QC samples were injected at the beginning and end of the batch to ensure system equilibration. Furthermore, sample acquisition was randomised, and the QC sample analysed every 10 injections to monitor and correct changes in the instrument response. 

### 3.4. Data Processing and Multivariate Data Analysis

The acquired raw datasets were processed using XCMS Online (http://XCMSOnline.scripps.edu/ (accessed on 22 July 2021). The data was processed with the following HPLC/UHD-qTOF parameters: feature detection was performed using the centWave method, maximal tolerated *m/z* was set to 5 ppm, signal to noise ratio was set to 7, the prefilter intensity and noise filter were set to 700 and 15 respectively. The retention time correction was performed using the obiwarp method with a profStep of 0.5. other parameters were set as bandwith = 0.5, minfrac (minimum fraction of sample in a group to be referred to as a feature) and mzwid (*m/z* width to determine peak groupings) of 0.025. The Kruskal–Wallis non-parametic method was used to perform the statistical test, post-hoc analysis was also performed, and the data was normalized using the median fold change. The resulting feature table with 3393 features was imported into SIMCA (soft independent modeling of class analogy) version 17.0 software (Sartorius, South Africa). The imported data was scaled using the square root of the standard deviation by applying *Pareto*-scaling [55]. The models presented in this study are principal component analysis (PCA) and hierarchical cluster analysis (HCA) of which the Ward’s method was used to calculate the distance. This are exploratory unsupervised models that assess the intrinsic structure of a dataset highlighting trends/patterns within a dataset [56].

### 3.5. Molecular Networking and Metabolite Annotation

Molecular networks were constructed on the GNPS website (http://gnps.ucsd.edu (accessed on 30 August 2021) using the online workflow (https://ccms-ucsd.github.io/GNPSDocumentation/ (accessed on 14 September 2021). Raw data obtained from the Shimadzu LCMS-9030 qTOF was converted to an open-source format (.mzML) prior to being uploaded to the online workflow. Once uploaded the data was processed by filtering and removing all MS^2^ fragment ions within +/− 17 Da of the precursor *m*/*z*. MS/MS spectra were window filtered by choosing only the top 6 fragment ions in the +/− 50 Da window throughout the spectrum. The data was clustered using an MS-CLUSTER algorithm. The precursor ion mass tolerance and the MS^2^ fragment ion were set to 0.05 Da. Edges (interconnections between metabolites/similarity) were formed only if the minimum cosine score of 0.7 was exceeded with more than 6 matched peaks. The network TopK was set to 10 of which only nodes in each other’s respective top 10 similar nodes were kept in the network. The maximum number of nodes that can be connected into a single molecular family was set 100 and the lowest scoring edges were removed. Spectra in the network were searched across spectral databases such as MassBank, ReSpect and NIST where the library spectra were filtered in the same manner as the sample spectra. The generated molecular networks were visualised using Cytoscape software [23,38]. All matched and some unmatched nodes were verified or putatively annotated using their empirical formulars generated from accurate mass and fragmentation patterns obtained from MS^2^ experiments. These were also searched against some common dereplication databases for natural products such as KNApSAck (http://www.knapsackfamily.com/knapsack_core/top.php (accessed on 9 October 2021), ChemSpider (http://www.chemspider.com/ (accessed on 17 September 2021), PubChem (https://pubchem.ncbi.nlm.nih.gov/ (accessed on 9 September 2021) and Dictionary of Natural Products (http://dnp.chemnetbase.com/faces/chemical/ChemicalSearch.xhtml (accessed on 7 September 2021). These were also compared to available literature. Metabolite annotation was carried out at level 2 of the Metabolomics Standards Initiative (MSI) [57]. The network generated was further explored using network annotation propagation (NAP), in which in silico fragmentation was performed and structural searches were performed in databases such as GNPS, HMDB, SUPNAT, CHEBI, DRUGBANK, FooDB. All ions were set to be deprotonated, and both the consensus score and fusion scores were calculated based on the first 10 candidates. The molecular network was also explored using Dereplicator for peptidic structural annotation. Substructure annotation was performed using MS2LDA interface in GNPS. Rhamnaceae, MassBank, GNPS and Urine Mass2Motifs were included in the search. The parameters were set as follows: overlap score threshold of 0.3, probability score of 0.1 and TopX of 5.

### 3.6. Pathway Analysis and Relative Quantification

Annotated metabolites, with available KEGG IDS were used for pathway analysis using the MetPA (metabolomic pathway analysis) tool, which is a web-based tool in MetaboAnalyst (version 5). KEGG IDs were used as input for pathway analysis, with the following parameters: the hypergeometric test was used as the enrichment method for the over overrepresentation analysis, relative betweenness-centrality was chosen for the node importance measure/topological analysis, scatterplots were used for the visualisation and *Arabidopsis thaliana* (thale cress) (KEGG) was chosen as the pathway library. Relative quantification was performed by computing colour-coded heatmaps in MetaboAnalyst, where peak intensity table was imported, the uploaded data was normalised, Pareto-scaled and log-transformed. Other parameters that were set included use of the Ward’Ss clustering algorithm and Pearson’s correlation. To simplify the visualisation of the abundances of the metabolites across the species, the top 25 metabolites ranked by t-test or ANOVA are shown (Figure 4D).

## 4. Conclusions

The use of computational tools to unravel the metabolomes of *M. balsamina, M. charantia, M. cardiospermoides,* and *M. foetida* permitted the semi-automated identification of known metabolites through spectral library matching, whilst insight derived MNs assisted in identification of other structurally related molecules. This led to the putative annotation of some metabolites that were not previously described in the *Momordica* genus. This study also led to the first metabolite profiling of *M. cardiospermoides*, which showed some significant chemotaxonomic differences to *M. charantia*, *M. foetida*, and *M.balsamina* especially with regard to the presence of some unique saponins. The phytochemical diversity of these plants potentially indicates the relevance of these plants for nutrition and as rich sources of natural products. The application of molecular networking and a combination of other computational tools in the GNPS ecosystem was shown to hold great potential in unravelling complex plant metabolomes, thus filling knowledge gaps of both unexplored and partially explored metabolomes. Future perspectives will involve further mining of these metabolomes using more computational tools to discover novel compounds that could be potential natural products, and to describe approaches that will assist in a more automated exploration of complex metabolomes.

## Data Availability

Not applicable.

## Supplementary Materials

The following are available online at https://www.mdpi.com/article/10.3390/metabo11110763/s1, Network analysis, in silico annotation and substructure annotation job links, Figure S1: Representative base peak intensity chromatograms (BPC) indicating the separation of metabolites in methanol extracts of *M. balsamina* (A), *M. cardiospermoides* (B), *M. charantia* (C) and *M. foetida*, Figure S2: Principal component analysis (PCA) and HCA of ESI negative data of *M. balsamina*, *M. Charantia*, *M. foetida* and *M. Cardiospermoides*, Figure S3: Molecular Network (A) of *M. balsaima, M. charantia, M. foetida and M. cardiospermoides* for spectral data (ESI negative mode). (B) MolNetEnhancer output showing different super classes within the four species, Table S1: Profiling of metabolites in *Momordica balsamina, Momordica cardiospermoides*, *Momordica charantia* and *Momordica Foetida* reported to level 2 or 3 of the Metabolomics standards initiative [51]. These metabolites were annotated from ESI (−) and ESI (+) data of the methanol extracts of the four *Momordica* species, Table S2: Pathway analysis of metabolites identified in the four *Momordica* species.
