# Supplementary material for: Metabolomics and Molecular Networking to Characterize the Chemical Space of Four Momordica Plant Species
Source: Metabolites. 2021 Nov 8;11(11):763. doi: 10.3390/metabo11110763 (PMC8619687; doi:10.3390/metabo11110763)
Supplement: Supplementary file 1 [file metabolites-11-00763-s001.zip › metabolites-1418225-supplementary.pdf]

metabolites

MDPI

Type of the Paper (Supplementary)

1. Network analysis, in silico annotation and substructure annotation job links

Molecular network ESI(-): https://gnps.ucsd.edu/ProteoSAFe/status.jsp?task=8cbf62e1396c4008bad70ebf22254683

Network propagation annotation ESI(-): https://proteomics2.ucsd.edu/ProteoSAFe/status.jsp?task=6a7f35441ed04f98b0d2b83471a4f762

DEREPLICATOR ESI(-): https://gnps.ucsd.edu/ProteoSAFe/status.jsp?task=35e70a6bac1c4bc88e25b41c93010bce

MS2LDA ESI(-): https://gnps.ucsd.edu/ProteoSAFe/status.jsp?task=7c498c0448724335845f2f72e85b1c20

MolNetEnhancer ESI (-): https://gnps.ucsd.edu/ProteoSAFe/status.jsp?task=700d9c1608774c47bc4d9961420c01da

Molecular network ESI(+): https://gnps.ucsd.edu/ProteoSAFe/status.jsp?task=2abe21d19056482da10e5150e969d621

Network propagation annotation ESI(+): https://proteomics2.ucsd.edu/ProteoSAFe/status.jsp?task=441f7a37e2a249c680bf5fd9d527f9fc

DEREPLICATOR ESI(+): https://gnps.ucsd.edu/ProteoSAFe/status.jsp?task=35e70a6bac1c4bc88e25b41c93010bce

MS2LDA ESI(+): https://gnps.ucsd.edu/ProteoSAFe/status.jsp?task=ff8361dd58c14848bcdfb0647ce7c215

MolNetEnhancer ESI (+): https://gnps.ucsd.edu/ProteoSAFe/status.jsp?task=8cfa702d7def4b72a108abbf9bade39f

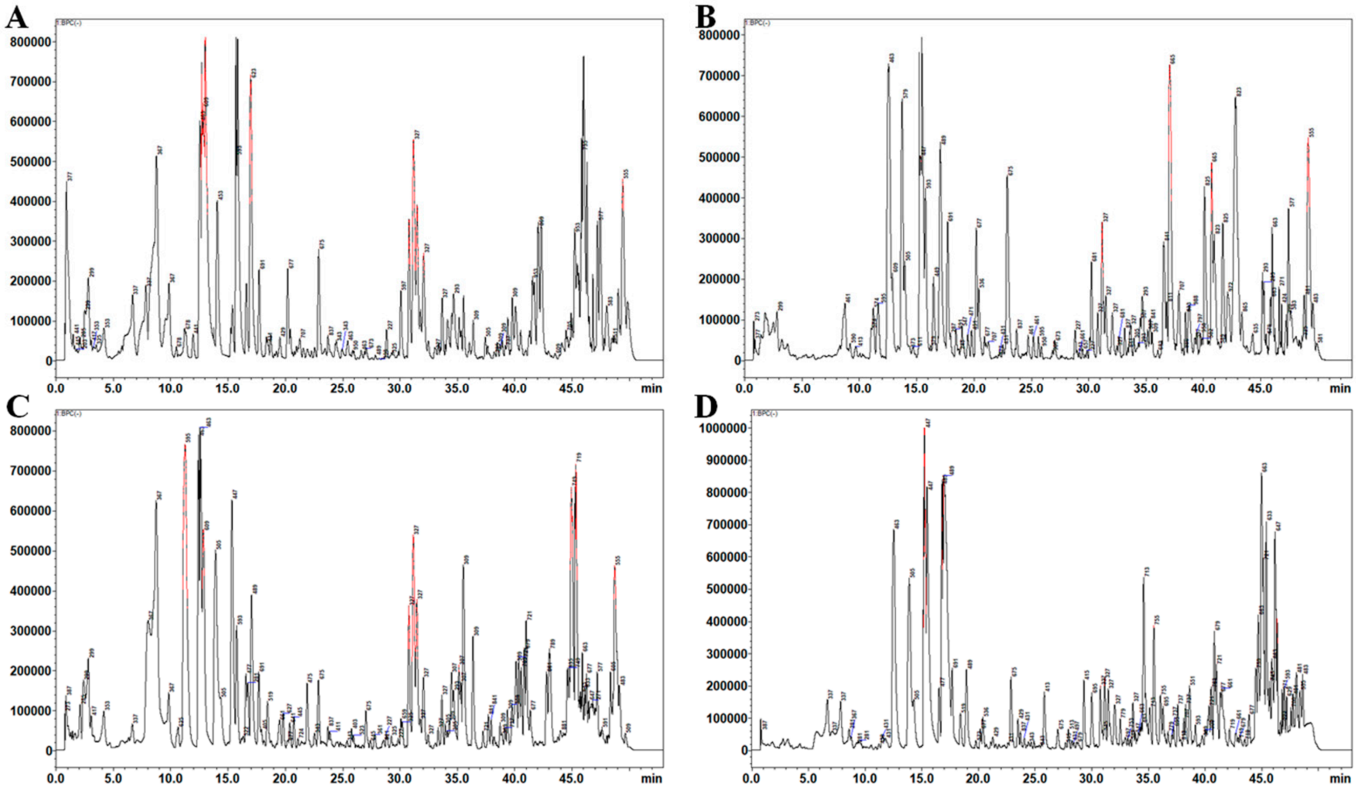

Figure S1: Representative base peak intensity chromatograms (BPC) indicating the separation of metabolites in methanol extracts of *M. balsamina* (A), *M. cardiospermoides* (B), *M. charantia* (C) and *M. foetida* indicating some differences in the metabolite profiles of these species. Hereby these species are depicted as phytochemically rich plants containing a wide variety of metabolites.

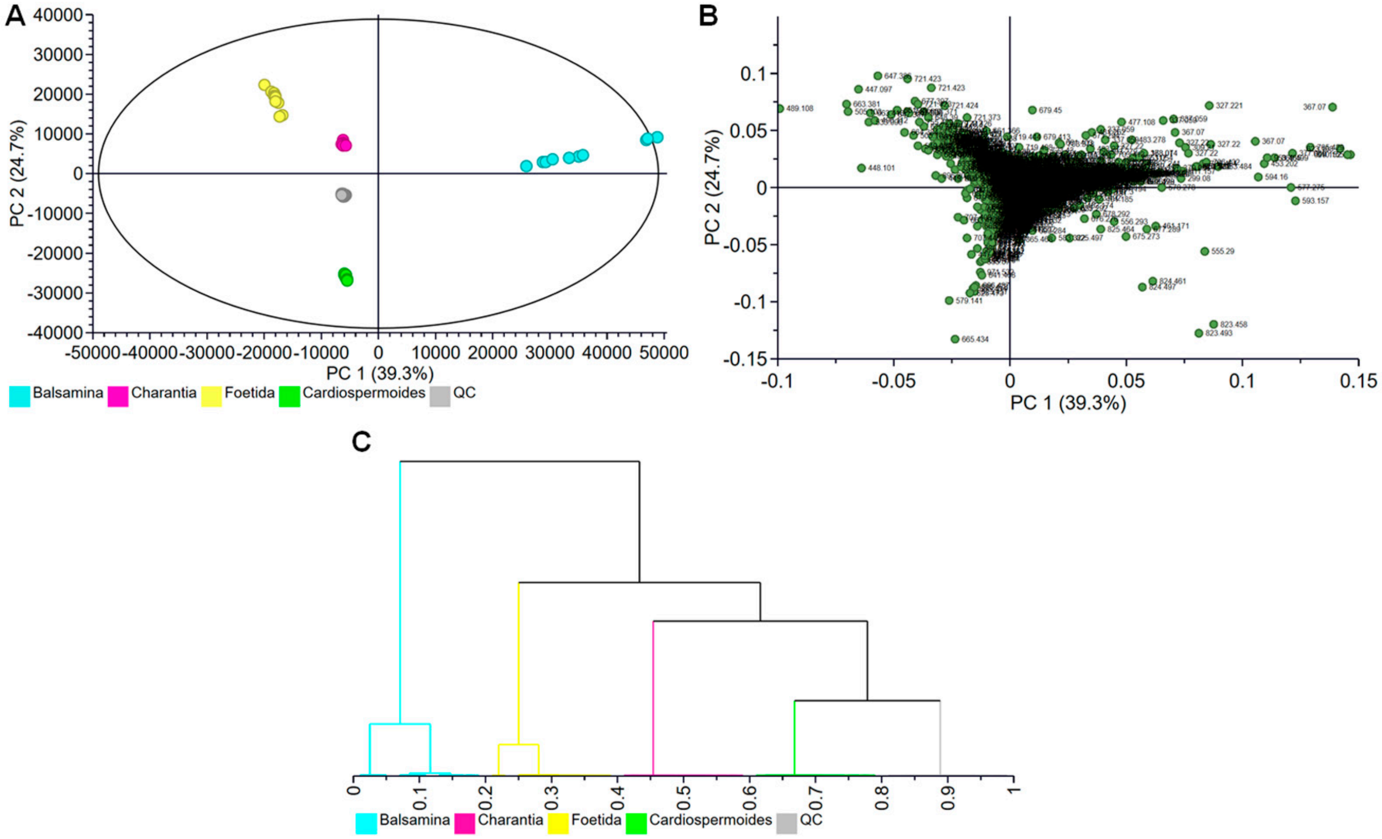

Figure S2: Unsupervised analysis of ESI negative data of *M. balsamina*, *M. Charantia*, *M. foetida* and *M. Cardiospermoides*. A PCA scores plot of the Pareto-scaled dataset of the four species including the quality control samples. The generated model was a 6-component model with PC 1 and PC 2 explaining 64% of the variation (A). The PCA loadings plot showing metabolites (outside the square) that contributed to the intrinsic patterns observes in the data set indicated in the scores plot (B). The HCA dendogram indicating the hierarchical structure of the dataset, highlighting some species-specific metabolite differences (B).

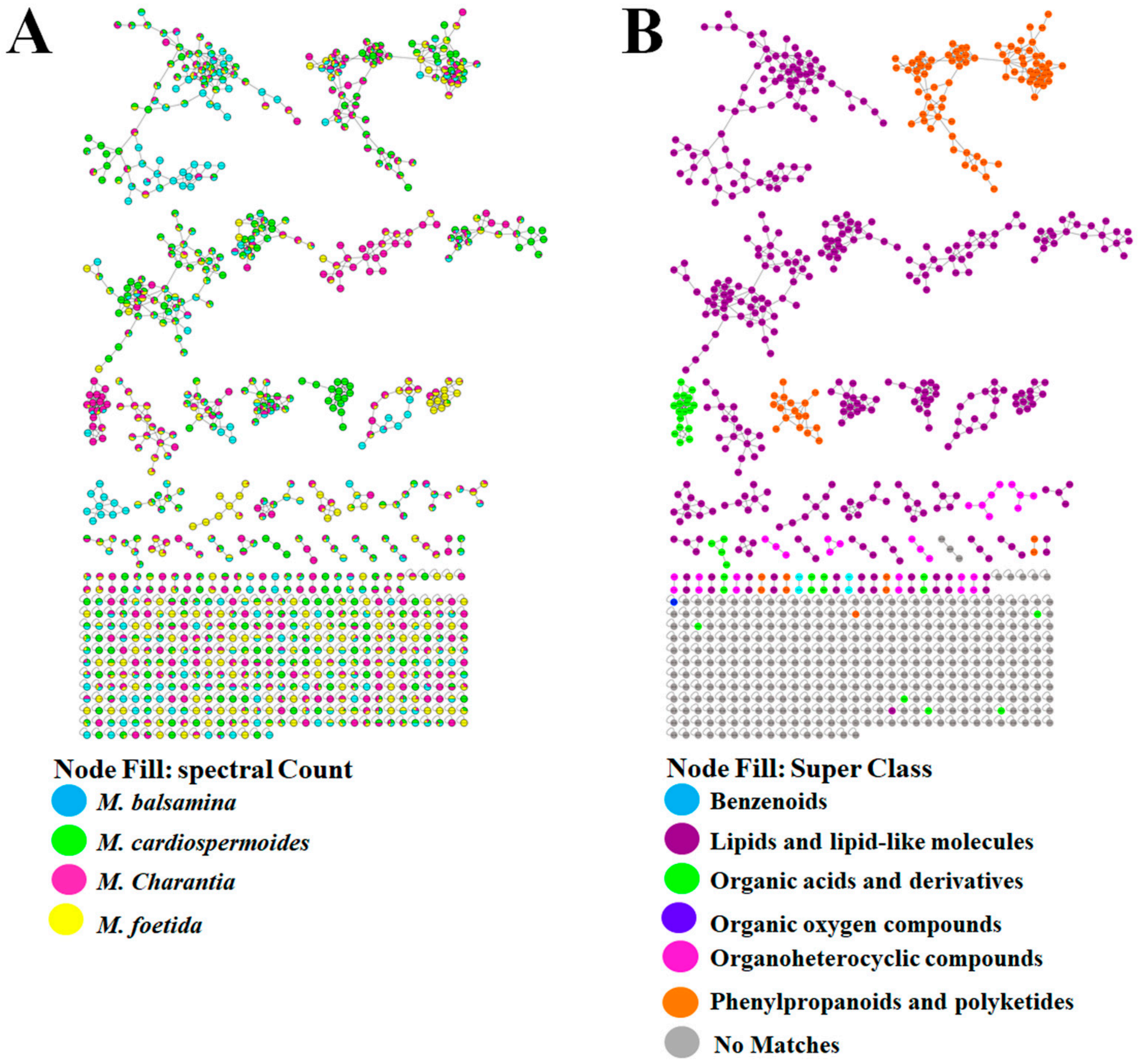

Figure S3: Molecular Network (A) of *M. balsaima, M. charantia, M. foetida and M. cardiospermoides* for data generated using a liquid chromatography-tandem mass spectrometry using electrospray ionisation in negative mode. (B) MolNetEnhancer output showing different super classes within the four species.

metabolites

MDPI

Table S1: Profiling of metabolites in *Momordica balsamina, Momordica cardiospermoides*, *Momordica charantia* and *Momordica Foetida* reported to level 2 or 3 of the Metabolomics standards initiative [51]. These metabolites were annotated from ESI (-) and ESI (+) data of the methanol extracts of the four *Momordica* species. Distribution of metabolites across the species is indicated by ✓ (presence) and × (absence).

No.	*m/z*	Adduct	Rt	Elemental composition	Fragment ions	Putative annotation	Superclass	Class	Subclass	*M.balsamina*	*M. cardiospermoides*	*M. charantia*	*M. foetida*
1	191.0216	$[M-H]^-$	1.02	$C_6H_8O_7$	111	Isocitric acid	Organic acids and derivatives	Carboxylic acids and derivatives	Tricarboxylic acids and derivatives	✓	✓	✓	✓
2	328.1366	$[M+H]^+$	1.08	$C_{15}H_{21}NO_7$	264, 166, 132	*N*-(1-deoxy-1-fructosyl)phenylalanine	Organic acids and derivatives	Carboxylic acids and derivatives	Amino acids	✓	✓	×	✓
3	367.1472	$[M+H]^+$	1.16	$C_{17}H_{22}N_2O_7$	229,188	Tryptophan N-glucoside	Carbohydrates	Glycosides	glucosides	✓	✓	×	✓
4	195.0522	$[M-H]^-$	1.18	$C_6H_{12}O_7$	191	Gluconic acid	Organic oxygen compounds	Organooxygen compounds	Carbohydrates and carbohydrate conjugates	✓	✓	✓	✓
5	164.0728	$[M-H]^-$	1.38	$C_9H_{11}NO_2$	147	Phenyalanine	Organic acids and derivatives	Carboxylic acids and derivatives	Amino acids	✓	✓	✓	✓
6	133.0150	$[M-H]^-$	1.40	$C_4H_6O_5$	115	Malic acid	Organic acids and derivatives	Hydroxy acids and derivatives	Carboxylic acids	✓	✓	✓	✓
7	315.0746	$[M-H]^-$	1.50	$C_{13}H_{16}O_9$	153, 152, 109, 108	2,5-Dihydroxybenzoic acid	Benzenoids	Benzene and substituted derivatives	Benzoic acids and derivatives	✓	✓	✓	✓
8	203.0842	$[M-H]^-$	1.74	$C_{11}H_{12}N_2O_2$	142, 116	Tryptophan	Organic acids and derivatives	Carboxylic acids and derivatives	Amino acid	✓	✓	✓	✓
9	137.0252	$[M-H]^-$	2.75	$C_7H_6O_3$	-	Hydroxybenzoic acid	Benzenoids	Benzene and substituted derivatives	Benzoic acids and derivatives	✓	✓	✓	✓
10	299.0793	$[M-H]^-$	2.95	$C_{13}H_{16}O_8$	137	Hydroxybenzoic acid glucoside	Benzenoids	Benzene and substituted derivatives	Benzoic acids and derivatives	✓	✓	✓	✓

11	565.1519	$[M+H]^+$	3.20	$C_{26}H_{28}O_{14}$	303	Kaempferol 3-rhamnoside-7-xyloside	Phenylpropanoids and polyketides	Flavonoids	Flavonoid glycosides	×	×	✓	×
12	353.0541	$[M-H]^-$	3.81	$C_{15}H_{14}O_{10}$	191,155, 111	5-Caffeoylisocitric acid	Phenylpropanoids and polyketides	Cinnamic acids and derivatives	Hydroxycinnamic acids and derivatives	✓	×	✓	×
13	195.0633	$[M+H]^+$	4.06	$C_{10}H_{10}O_4$	149, 117	Ferulic acid	Phenylpropanoids and polyketides	Cinnamic acids and derivatives	Hydroxycinnamic acids and derivatives	✓	×	✓	✓
14	355.1061	$[M-H]^-$	5.10	$C_{16}H_{20}O_9$	193, 179, 163, 134	Feruloyl-D-glucose	Phenylpropanoids and polyketides	Cinnamic acids and derivatives	Hydroxycinnamic acids and derivatives	✓	×	×	×
15	303.0478	$[M+H]^+$	5.13	$C_{15}H_{10}O_7$	257. 153	Quercetin	Phenylpropanoids and polyketides	Flavonoids	Flavanols	✓	✓	✓	✓
16	163.0416	$[M-H]^-$	5.21	$C_9H_8O_3$	119	Coumaric acid	Phenylpropanoids and polyketides	Cinnamic acids and derivatives	Hydroxycinnamic acids and derivatives	✓	✓	✓	✓
17	465.0989	$[M+H]^+$	5.24	$C_{21}H_{20}O_{12}$	303	Quercetin glucoside, Isoquercitrin, Spiraeoside 1	Phenylpropanoids and polyketides	Flavonoids	Flavonoid glycosides	✓	✓	✓	✓
18	595.1703	$[M-H]^-$	5.43	$C_{27}H_{32}O_{15}$	475, 415, 385, 355, 313	6,8-di-C-glucopyranosylnaringenin	Phenylpropanoids and polyketides	Flavonoids	Flavonoid glycosides	✓	×	✓	✓
19	287.0530	$[M+H]^+$	5.87	$C_{15}H_{10}O_6$	153	Kaempferol	Phenylpropanoids and polyketides	Flavonoids	Flavanols	✓	✓	✓	✓
20	595.1614	$[M+H]^+$	6.51	$C_{27}H_{30}O_{15}$	449, 287	7-[4,5-dihydroxy-6-(hydroxymethyl)-3-[(2S,3R,4R,5R,6S)-3,4,5-trihydroxy-6-methyloxan-2-yl]oxyoxan-2-yl]oxy-2-(3,4-	Phenylpropanoids and polyketides	Flavonoids	Flavonoid glycosides	×	✓	✓	×

						dihydroxy-phenyl)-5-hydroxy-chromen-4-one							
21	385.1167	$[M-H]^-$	7.10	$C_{17}H_{22}O_{10}$	223, 298, 179, 164, 179	Sinapoyl glycoside	Organooxygen compound	Carbohydrates and carbohydrate derivative	Carbohydrate derivative	✓	✓	×	×
22	337.0592	$[M-H]^-$	7.63	$C_{15}H_{14}O_9$	173, 154, 111	4-Coumaroyl-isocitric acid	Phenylpropanoids and polyketides	Cinnamic acids and derivatives	Hydroxycinnamic acids and derivatives	✓	×	✓	✓
23	609.1528	$[M-H]^-$	8.35	$C_{27}H_{31}O_{16}^+$	447, 285	Cyanidin-3, 5-di-O-glucoside	Phenylpropanoids and polyketides	Flavonoids	Flavonoid glycosides	×	✓	×	×
24	367.0702	$[M-H]^-$	8.65	$C_{16}H_{16}O_{19}$	173, 154, 111	4-Feruloyl-isocitric acid	Phenylpropanoids and polyketides	Cinnamic acids and derivatives	Hydroxycinnamic acids and derivatives	✓	×	✓	×
25	563.1455	$[M-H]^-$	11.10	$C_{26}H_{28}O_{14}$	545, 531, 443, 353,	Apigenin 6-C-glucoside 8-C-arabinoside	Phenylpropanoids and polyketides	Flavonoids	Flavonoid glycosides	×	✓	×	×
26	595.1324	$[M-H]^-$	11.27	$C_{26}H_{22}O_{16}$	300	Quercetin pentosylhexoside/ quercetin-3-O-arabinoglucoside	Phenylpropanoids and polyketides	Flavonoids	Flavonoid glycosides	×	✓	×	✓
27	463.0898	$[M-H]^-$	12.31	$C_{12}H_{20}O_{12}$	300	Quercetin glucoside, Isoquercitrin, Spiraeoside 2	Phenylpropanoids and polyketides	Flavonoids	Flavonoid glycosides	✓	✓	✓	✓
28	609.2680	$[M-H]^-$	12.89	$C_{27}H_{30}O_{16}$	300	Quercetin rutinoside 1/ rutin	Phenylpropanoids and polyketides	Flavonoids	Flavonoid glycosides	✓	✓	×	✓
29	609.1523	$[M-H]^-$	13.07	$C_{27}H_{30}O_{16}$	300	Quercetin rutinoside 2/ rutin	Phenylpropanoids and polyketides	Flavonoids	Flavonoid glycosides	✓	✓	×	✓
30	579.138	$[M-H]^-$	13.46	$C_{26}H_{22}O_{15}$	284	Kaempferol-3-*O*-hexose-*O*-pentoside	Phenylpropanoids and polyketides	Flavonoids	Flavonoid glycosides	×	✓	×	×

31	505.1002	$[M-H]^-$	13.59	$C_{23}H_{21}O_{13}$	300	Quercetin acetyl hexose 1	Phenylpropanoids and polyketides	Flavonoids	Flavonoid glycosides	×	✓	✓	✓
32	579.1408	$[M-H]^-$	13.76	$C_{26}H_{28}O_{15}$	284	Kaempferol 3-apiosyl-(1->2)-galactoside/Kaempferol 3-vicianoside 1	Phenylpropanoids and polyketides	Flavonoids	Flavonoid glycosides	×	✓	×	×
33	681.1365	$[M-H]^-$	13.78	$C_{29}H_{31}O_{19}+$	637, 300	Delphinidin 3-(6''-malonylsambubioside)	Phenylpropanoids and polyketides	Flavonoids	Flavonoid glycosides	×	✓	×	×
	549.1790	$[M-H]^-$		$C_{24}H_{22}O_{15}$	505, 300, 151	Quercetin-3-O-malonyl-glucoside1	Phenylpropanoids and polyketides	Flavonoids	Flavonoid glycosides	×	✓	✓	✓
34	579.1407	$[M-H]^-$	14.01	$C_{26}H_{28}O_{15}$	285	Kaempferol 7-apiosyl-(1->2)-galactoside/Kaempferol 7-vicianoside 2	Phenylpropanoids and polyketides	Flavonoids	Flavonoid glycosides	×	×	✓	×
35	549.0935	$[M-H]^-$	14.34	$C_{24}H_{22}O_{15}$	505, 300, 151	Quercetin-3-O-malonyl-glucoside1	Phenylpropanoids and polyketides	Flavonoids	Flavonoid glycosides	✓	✓	✓	✓
36	505.1033	$[M-H]^-$	14.9	$C_{23}H_{21}O_{13}$	300	Quercetin acetyl hexose 2	Phenylpropanoids and polyketides	Flavonoids	Flavonoid glycosides	✓	✓	✓	✓
37	609.1514	$[M-H]^-$	15.29	$C_{27}H_{30}O_{16}$	315	Isorhamnetin 3-glucoside-7-alpha-L-arabinopyranoside	Phenylpropanoids and polyketides	Flavonoids	Flavonoid glycosides	×	✓	×	×
38	447.1080	$[M-H]^-$	15.43	$C_{21}H_{20}O_{11}$	284, 255,227, 151	Kaempferol glucoside 1	Phenylpropanoids and polyketides	Flavonoids	Flavonoid glycosides	✓	✓	✓	✓
39	549.0931	$[M-H]^-$	16.23	$C_{24}H_{22}O_{15}$	505, 300	Quercetin3-O-malonyl-glucoside2	Phenylpropanoids and polyketides	Flavonoids	Flavonoid glycosides	✓	✓	✓	✓
40	447.0995	$[M-H]^-$	16.61	$C_{21}H_{20}O_{11}$	284, 255,227, 151	Kaempferol glucoside 2	Phenylpropanoids and polyketides	Flavonoids	Flavonoid glycosides	✓	✓	✓	✓

41	665.1470	[M-H]$^-$	15.47	$C_{29}H_{30}O_{18}$+	621, 579, 621, 284	Cyanidin 3-(6''-malonylsambubioside)	Phenylpropanoids and polyketides	Flavonoids	Flavonoid glycosides	×	✓	×	×
42	593.1537	[M-H]$^-$	15.87	$C_{27}H_{30}O_{15}$	285	Kaempferol rutinoside	Phenylpropanoids and polyketides	Flavonoids	Flavonoid glycosides	✓	✓	✓	×
43	477.1078	[M-H]$^-$	16.59	$C_{22}H_{22}O_{12}$	314	Isorhamnetin-3-O-glucoside	Phenylpropanoids and polyketides	Flavonoids	Flavonoid glycosides	✓	×	✓	✓
44	623.1675	[M-H]$^-$	16.98	$C_{28}H_{32}O_{16}$	315	Isorhamnetin rutinoside	Phenylpropanoids and polyketides	Flavonoids	Flavonoid glycosides	✓	×	×	×
45	489.1056	[M-H]$^-$	17.1	$C_{23}H_{22}O_{11}$	284, 255, 277, 151	Kaempferol acetyl hexose	Phenylpropanoids and polyketides	Flavonoids	Flavonoid glycosides	×	✓	✓	✓
46	533.0982	[M-H]$^-$	17.39	$C_{24}H_{22}O_{14}$	489, 285, 255	Kaempferol malonylglucoside 1	Phenylpropanoids and polyketides	Flavonoids	Flavonoid glycosides	✓	✓	✓	✓
47	449.1121	[M-H]$^-$	18.01	$C_{21}H_{22}O_{11}$	287	Isookanin-7-glucoside	Phenylpropanoids and polyketides	Flavonoids	Flavonoid glycosides	✓	✓	×	✓
48	533.0980	[M-H]$^-$	18.87	$C_{24}H_{22}O_{14}$	489, 285, 255	Kaempferol malonylglucoside 2	Phenylpropanoids and polyketides	Flavonoids	Flavonoid glycosides	✓	×	✓	✓
49	431.1024	[M-H]$^-$	18.38	$C_{21}H_{20}O_{10}$	284, 255, 227, 163	Kaempferol 3-O-rhamnoside/ Afzelin	Phenylpropanoids and polyketides	Flavonoids	Flavonoid glycosides	✓	×	×	×
50	519.1185	[M-H]$^-$	18.65	$C_{24}H_{24}O_{13}$	314	Isorhamnetin acetylglucoside	Phenylpropanoids and polyketides	Flavonoids	Flavonoid glycosides	✓	✓	✓	×
51	437.3382	[M+H]$^+$	19.25	$C_{30}H_{44}O_2$		Cucurbita-5,23,25-triene-3,7-dione	Lipid and lipid like molecules	Prenol lipids	Triterpenoid	×	✓	×	×
52	471.3433	[M+H]$^+$	19.56	$C_{30}H_{46}O_4$		Momordic acid/ Balsaminol C	Lipid and lipid like molecules	Prenol lipids	Triterpenoid	×	✓	✓	✓
53	573.1296	[M-H]$^-$	19.68	$C_{27}H_{26}O_{14}$	285	6,7,3',4'-Tetrahydroxyaurone 6-	Phenylpropanoids and polyketides	Flavonoids	Flavonoid glycosides	×	×	×	✓

						(2'',4'',6''-tri-acetylglucoside)/Maritimetin 6-O-(3'',4'',6''-tri-O-acetylglucoside)						
54	399.1333	$[M-H]^-$	19.69	$C_{18}H_{24}O_{10}$	163, 145	Regaloside A	Phenylpropanoids and polyketides	Cinnamic acids and derivatives	Hydroxycinnamic acids and derivatives	×	×	✓	×
55	455.3480	[M+H]+	20.33	$C_{30}H_{46}0_3$		Charantadiol A	Lipid and lipid like molecules	Prenol lipids	Triterpenoid	×	✓	✓	×
56	601.4073	[M+H]+	21.82	$C_{36}H_{56}O_7$		Charantoside IV/III	Lipid and lipid like molecules	Prenol lipids	Triterpenoid	×	✓	×	×
57	585.4103	[M+H]+	22.37	$C_{36}H_{56}O_6$		Triacetylbalsaminol	Lipid and lipid like molecules	Prenol lipids	Triterpenoid	✓	✓	✓	✓
58	455.3484	$[M+H]^+$	25.16	$C_{30}H_{46}O_3$	133, 119	3-Hydroxy-11-ursen-28,13-olide	Lipid and lipid like molecules	Prenol lipids	Triterpenoid	×	✓	✓	×
59	457.1479	$[M+H]^+$	25.89	$C_{30}H_{48}O_3$		Balsaminol E	Lipid and lipid like molecules	Prenol lipids	Triterpenoid	×	✓	×	×
60	439.3570	[M+H]	29.57	$C_{30}H_{46}O_2$		Momordicinin	Lipid and lipid like molecules	Prenol lipids	Triterpenoid	✓	✓	✓	✓
61	695.4081	$[M-H]^-$	30.00	$C_{37}H_{60}O_{12}$		Momordicoside E	Lipid and lipid like molecules	Prenol lipids	Triterpenoid	×	×	×	✓
62	677.4301	[2M-2H+Na]	31.18	$C_{18}H_{32}O_5$	327	(10E,15E)-9,12,13-trihydroxyoctadeca-10,15-dienoic acid	Lipid and lipid like molecules	Prenol lipids	Fatty acyl	✓	✓	✓	×
63	327.2203	$[M-H]^-$	31.83	$C_{18}H_{32}O_4$	171, 239	FA 18:2+2O	Lipid and lipid like molecules	Prenol lipids	Fatty acid	✓	✓	✓	✓
64	454.2896	$[M+H]^+$	34.52	$C_{21}H_{44}NO_7P$	313	1-Palmitoyl-2-hydroxy-sn-glycero-3-	Lipid and lipid like molecules	Glycerophosphoethanolamines	Glycerophospholipid	✓	✓	✓	✓

						phosphoethanolamine							
65	496.4062	$[M+H]^+$	35.57	$C_{25}H_{54}NO_6P$	478, 313, 184, 104	1-O-Hexadecyl-2-C-methyl-3-phosphatidylcholine	Lipid and lipid like molecules	Glycerophosphocholines	Glycerophospholipids	✓	✓	✓	✓
66	841.4644	[M+FA-H]	36.52	$C_{41}H_{64}O_{15}$	795, 633	Diginatin/1-O-[(2beta,3beta,5xi,9xi,18xi)-2,23-Dihydroxy-28,29-dioxo-3-(beta-D-xylopyranosyloxy)olean-12-en-28-yl]-beta-D-glucopyranose	Lipid and lipid like molecules	Steroidal glycosides	Triterpenoids	×	×	×	✓
67	522.3524	$[M+H]^+$	36.64	$C_{26}H_{52}NO_7P$	522, 504, 184	1-(9Z-Octadecenoyl)-sn-glycero-3-phosphocholine	Lipid and lipid like molecules	Glycerophosphocholines	Glycerophospholipids	×	✓	✓	×
68	713.4168	$[M-H]^-$	36.85	$C_{37}H_{62}O_{13}$	505, 575,179	Cyclopassifloside VII	Lipid and lipid like molecules	Prenol lipids	Terpene glycosides	×	×	×	✓
69	757.4440	[M+FA-H]	36.85	$C_{38}H_{58}O_{15}$		NCGC00347358-02_C38H58O15_Card-20(22)-enolide, 1-(acetyloxy)-3-[[(1R)-4-O-(6-deoxy-3-O-methylhexopyra-	Lipid and lipid like molecules	Prenol lipids	Fatty acid	✓	×	✓	✓

						nosyl)hex-opyra-nosyl]oxy]-14-hydroxy-, (1beta,3beta, 8xi,9xi)-							
70	779.4178	[M-H]$^-$	37.46	$C_{41}H_{64}O_{14}$		Cynarasaponin I/F	Lipid and lipid like molecules	Prenol lipids	Terpene glycosides	✗	✗	✗	✓
71	939.5057	[M-H]$^-$	38.68	$C_{48}H_{76}O_{18}$		Momordin IIa	Lipid and lipid like molecules	Prenol lipids	Terpene glycosides	✗	✓	✗	✗
72	681.4162	[M-H]$^-$	38.94	$C_{37}H_{62}O_{11}$		Cyclopassifloside II	Lipid and lipid like molecules	Prenol lipids	Terpene glycosides	✓	✓	✗	✗
73	811.5127	M+HCOO	38.89	$C_{43}H_{72}O_{14}$	765, 661, 619	Ginsenoside Rg6	Lipid and lipid like molecules	Prenol lipids	Terpene glycosides	✗	✓	✗	✗
74	741.4494	[M+FA-H]	39.35	$C_{35}H_{52}O_{14}$	695, 533,363, 179	Xysmalorin/ NCGC00385195-01_C35H52O14_Carda-5,20(22)-dienolide, 3-[(2-O-beta-D-glucopyranosyl-beta-D-glucopyranosyl)oxy]-14-hydroxy-, (3beta)-	Lipid and lipid like molecules	Prenol lipids	Terpene glycosides	✗	✗	✗	✓
75	825.4726	[M-H]$^-$	40.13	$C_{43}H_{70}O_{15}$		Trojanoside A/ Astragaloside II	Lipid and lipid like molecules	Prenol lipids	Terpene glycosides	✓	✓	✗	✗
76	841.4526	[M-H]$^-$	40.25	$C_{43}H_{70}O_{16}$		Goyaglycoside-g	Lipid and lipid like molecules	Prenol lipids	Terpene glycosides	✓	✗	✓	✓

77	524.3675	[M+H]+	40.49	$C_{26}H_{54}NO_7P$	506, 447, 341, 258, 184, 104	1-Octadecanoyl-sn-glycero-3-phosphocholine	Lipid and lipid like molecules	Glycerophosphocholines	Glycerophospholipid	×	✓	✓	✓
78	697.4214	[M-H]-	41.11	$C_{37}H_{62}O_{12}$		Cyclopassifloside VIII/IV, X	Lipid and lipid like molecules	Prenol lipids	Terpene glycosides	×	×	×	✓
79	827.4794	[M-H]-	41.31	$C_{43}H_{72}O_{15}$		Vina-ginsenoside R2	Lipid and lipid like molecules	Prenol lipids	Terpene glycosides	×	✓	×	×
80	843.4683	[M-H]-	41.81	$C_{43}H_{72}O_{16}$	179	Cyclotricuspidoside A/ Cyclopassifloside III	Lipid and lipid like molecules	Prenol lipids	Terpene glycosides	×	×	✓	×
81	867.5450	[M-H]-	45.27	$C_{45}H_{72}O_{16}$		Astragaloside I/Isoastragaloside I	Lipid and lipid like molecules	Prenol lipids	Terpene glycosides	✓	×	×	×
82	552.3992	[M+H]+	45.69	$C_{28}H_{58}NO_7P$	258, 184	1-Arachidoyl-2-hydroxy-sn-glycero-3-phosphocholine	Lipid and lipid like molecules	Glycerophosphocholines	Glycerophospholipids	✓	✓	×	×
83	593.1460	[M+H]+	45.71	$C_{35}H_{36}N_4O_5$		Pheophorbide A	Organoheterocyclic compounds	Porphyrins	Tetrapyrroles	✓	×	✓	✓
84	721.4252	[M-H]-	46.03	$C_{33}H_{56}O_{14}$	277, 101	Glc-Glc-octadecatrienoyl-sn-glycerol	Lipid and lipid like molecules	Glycerophosphocholines	Fatty acid	✓	✓	✓	✓
85	881.5119	[M-H]-	46.86	$C_{47}H_{79}O_{13}P$		Massbank: LQB00300 PI 38:6 [1-Hexadecanoyloxy-3-[hydroxy-(2,3,4,5,6-pentahydroxycyclohexyl)oxyphos-	Lipid and lipid like molecules	Glycerophosphocholines	Glycerophospholipid	✓	×	×	✓

						phoryl]oxypropan-2-yl] (4Z,7Z,10Z,13Z,16Z,19Z)-docosa-4,7,10,13,16,19-hexaenoate							
86	599.326	[M-H]-	47.47	$C_{27}H_{53}O_{12}P$		1-Octadecanoyl-sn-glycero-3-phospho-(1'-myo-inositol)	Lipid and lipid like molecules	Glycerophosphocholines	Glycerophospholipid	✓	✗	✓	✓
87	511.4	[M-H]-	47.58	$C_{24}H_{49}O_9P$		1-Octadecanoyl-sn-glycero-3-phospho-(1'-sn-glycerol)	Lipid and lipid like molecules	Glycerophosphocholines	Glycerophospholipid	✗	✓	✓	✓
88	311.189	[M-H]-	47.60	$C_{18}H_{16}O_5$	297, 267, 253	Trimethoxyflavone	Phenylpropanoids and polyketides	Flavonoids	6-O-methylated flavonoids	✓	✗	✗	✗
89	747.525	[M-H]-	47.62	$C_{40}H_{77}O_{10}P$		1-Palmitoyl-2-oleoyl-sn-glycero-3-(phospho-rac-(1-glycerol))	Lipid and lipid like molecules	Glycerophosphocholines	Glycerophospholipid	✓	✗	✓	✗
90	673.477	[M-H]-	47.79	$C_{37}H_{71}O_8P$		(2R)-1-Hexadecanoyloxy-3-phosphonooxypropan-2-yl] (E)-octadec-9-enoate	Lipid and lipid like molecules	Glycerophosphocholines	Glycerophospholipid	✗	✗	✗	✓
91	782.5674	[M+H]+	47.90	$C_{44}H_{80}NO_8P$	184	1,2-Dilinoleoyl-sn-glycero-3-phosphocholine	Lipid and lipid like molecules	Glycerophosphocholines	Glycerophospholipids	✓	✗	✗	✓
92	835.5298	[M-H]-	47.95	$C_{43}H_{81}O_{13}P$	673, 595, 597,571, 533, 417	1-Hexadecanoyl-2-(9Z-	Lipid and lipid like molecules	Glycerophosphocholines	Glycerophospholipid	✗	✗	✗	✓

						octadecenoyl)-sn-glycero-3-phospho-(1'-myo-inositol)							
93	663.4499	[M+H]+	48.08	$C_{38}H_{62}O_9$		Goyaglycoside d	Lipid and lipid like molecules	Prenol lipids	Triterpenoid	✓	✓	✓	✓
94	701.461	[M-H]	48.10	$C_{39}H_{75}O_8P$		[3-[octadec-11-enoyloxy]-2-(octadecanoyloxy)propoxy]phosphonic acid	Lipid and lipid like molecules	Glycerophosphocholines	Glycerophospholipid	✓	×	✓	✓
95	325.1872	[M-H]-	48.50	$C_{22}H_{46}O$		Docosanol	Lipid and lipid like molecules	Fatty Acyls	Fatty alcohol	✓	✓	✓	✓
95	871.5692	[M+H]+	48.53	$C_{55}H_{74}N_4O_5$	593, 533	Pheophytin A	Lipid and lipid like molecules	Glycerophosphoethanolamines	Glycerophospholipid	×	✓	×	×
97	712.5421	[M-H]	48.71	$C_{40}H_{75}NO_9$	550	(+)-Soyacerebroside I	Lipid and lipid like molecules	sphingolipids	Glycosyl-n-acyl-sphingosines	✓	✓	✓	✓
98	760.5822	[M+H]+	48.57	$C_{42}H_{82}NO_8P$	184	1-Hexadecanoyl-2-(9Z-octadecenoyl)-sn-glycero-3-phosphocholine	Lipid and lipid like molecules	Glycerophosphocholines	Glycerophospholipid	×	×	✓	×
99	483.3442	[M-H]-	49.00	$C_{22}H_{45}O_9P$		1-Hexadecanoyl-sn-glycero-3-phospho-(1'-sn-glycerol)	Lipid and lipid like molecules	Glycerophosphocholines	Glycerophospholipid	✓	✓	✓	✓
100	571.358	[M-H]-	49.16	$C_{25}H_{49}O_{12}P$		1-Hexadecanoyl-sn-glycero-3-phospho-(1'-myo-inositol)	Lipid and lipid like molecules	Glycerophosphocholines	Glycerophospholipid	✓	✓	✓	✓

metabolites

Table S2: Pathway analysis of metabolites identified in the four *Momordica* species

No	Pathway	Total	Hits	*p*-value	Impact
1	Biosynthesis of secondary metabolites – unclassified	5	1	0.10964	1
2	Flavone and flavonol biosynthesis	10	5	1.09E-06	0.85
3	Phenylalanine metabolism	11	1	0.22586	0.47059
4	Glycerophospholipid metabolism	37	3	0.050294	0.19174
5	Glyoxylate and dicarboxylate metabolism	29	2	0.14109	0.16151
6	Pyruvate metabolism	22	1	0.40187	0.15462
7	Tryptophan metabolism	28	1	0.4808	0.12037
8	Phenylpropanoid biosynthesis	46	4	0.019014	0.0945
9	Citrate cycle (TCA cycle)	20	2	0.074932	0.07816
10	Sphingolipid metabolism	17	1	0.3273	0.0625
11	Carbon fixation in photosynthetic organisms	21	1	0.38763	0.05846
12	Inositol phosphate metabolism	28	1	0.4808	0.04393
13	Flavonoid biosynthesis	47	3	0.089942	0.0348
14	Porphyrin and chlorophyll metabolism	48	2	0.30201	0.02305
15	Phosphatidylinositol signaling system	26	1	0.45569	0.01559
16	Glycosylphosphatidylinositol (GPI)-anchor biosynthesis	13	1	0.26122	0.00476
17	Phenylalanine, tyrosine and tryptophan biosynthesis	22	2	0.088563	0.0015
18	Ubiquinone and other terpenoid-quinone biosynthesis	38	1	0.59044	0.00097
19	Arachidonic acid metabolism	12	2	0.029169	0
20	Linoleic acid metabolism	4	1	0.088687	0
21	Indole alkaloid biosynthesis	4	1	0.088687	0
22	Tropane, piperidine and pyridine alkaloid biosynthesis	8	1	0.16972	0
23	Anthocyanin biosynthesis	11	1	0.22586	0
24	Aminoacyl-tRNA biosynthesis	46	2	0.2847	0
25	Pentose phosphate pathway	19	1	0.35815	0
26	Biosynthesis of unsaturated fatty acids	22	1	0.40187	0
27	Glucosinolate biosynthesis	65	2	0.44436	0
28	alpha-Linolenic acid metabolism	28	1	0.4808	0
29	Cyanoamino acid metabolism	29	1	0.49294	0
30	Glycine, serine and threonine metabolism	33	1	0.53877	0
